# Analysis of genetic diversity and population structure of Moroccan date palm (*Phoenix dactylifera* L*.*) using SSR and DAMD molecular markers

**DOI:** 10.1186/s43141-023-00516-7

**Published:** 2023-05-23

**Authors:** Maha Ibrahimi, Najiba Brhadda, Rabea Ziri, Mohamed Fokar, Driss Iraqi, Fatima Gaboun, Mustapha Labhilili, Aicha Habach, Reda Meziani, Jamal Elfadile, Rabha Abdelwahd, Ghizlane Diria

**Affiliations:** 1 Biotechnology Unit, Regional Center of Agricultural Research of Rabat, National Institute of Agricultural Research (INRA), Rabat, Morocco; 2 Laboratory of Plant, Animal and Agro-Industry Productions, Faculty of Sciences, University of Ibn Tofail, Kenitra, Morocco; 3 Center for Biotechnology and Genomics, Texas Tech University, Lubbock, TX USA; 4 UR Oasis Systems, National Laboratory of Date Palm Tissues Culture, Regional Center of Agricultural Research of Errachidia, National Institute of Agricultural Research (INRA), Rabat, Morocco

**Keywords:** Genetic diversity, Date palm, SSR, DAMD, Structure populations, Climate change

## Abstract

**Background:**

Date palm, oasis pivot, plays a vital socio-economic part in the southern area of Morocco. However, with climate change and drought intensity and frequency increasing, the Moroccan palm grove is threatened with significant genetic degradation. Genetic characterization of this resource is key element for the development of effective conservation and management strategies in the current circumstances of climate change and various biotic and abiotic stresses. To evaluate the genetic diversity of date palm populations collected from different Moroccan oases, we used simple sequence repeats (SSR) and directed amplification of mini-satellite DNA (DAMD) markers. Our results showed that used markers could efficiently assess genetic diversity in *Phoenix dactylifera* L.

**Results:**

A total of 249 and 471 bands were respectively scored for SSR and DAMD, of which 100% and 92.9% were polymorphic. The polymorphic information content (PIC = 0.95), generated by the SSR primer was nearly identical to that generated by the DAMD primer (PIC = 0.98). The resolving power (Rp) was higher in DAMD than SSR (29.46 and 19.51, respectively). Analysis of the molecular variance (AMOVA) based on the combined data sets for both markers revealed a higher variance within populations (75%) than among populations (25%). Principal coordinate analysis (PCoA) and the ascendant hierarchical classification showed that the population of Zagora and Goulmima regions were the closest populations. The STRUCTURE analysis clustering of the 283 tested samples into seven clusters based on their genetic composition.

**Conclusion:**

The results drawn from this study will orient genotypes selection strategies for a successful future breeding and conservation program, particularly under climate change context.

**Supplementary Information:**

The online version contains supplementary material available at 10.1186/s43141-023-00516-7.

## Background

The date palm (*Phoenix dactylifera* L*.*) is a diploid (2*n* = 36), monocotyledonous plant [1]. It is a dioecious species and a member of the Arecaceae family [2]. It is one of the world's oldest plants, and has been farmed for over 6000 years, mainly for its fruit [3]. Dates are well-known for containing a complex mixture of nutritive and non-nutritive bioactive molecules. These include antioxidants [4], bioactive phytochemicals, water- and fat-soluble vitamins, non-starch polysaccharides, and minerals [5]. It is a key element in food security, and known as a “tree of life” [6].

*Phoenix dactylifera* L. significantly contributes towards the sustenance of the desert ecosystem. It combats desertification and modulates macro- and micro-climate temperatures. It also protects the soil from different types of degradation, promotes biodiversity, and reduces pollutants [7].

In Morocco, date palm represents about 4.5% of the world’s date population [8, 9]. Additionally, Morocco stands seventh in the world in area devoted to date-cultivation, eleventh in number of date palms, and ranks twelfth in the production of dates [8].

In 2018, the global production of dates reached 8.5 million tons (Mt) from a total cultivated area of 1.09 million hectares. For Morocco, the production is considered to be 1 million tons over an area of 59.127 ha [10].

Despite the availability of several cultivars, local date palm groves are seriously threatened by abiotic and biotic stresses such as, salinity, prolonged drought, and diseases [8] which could induce genetic degradation of this resource. In addition, the lack of data relating to the genetic diversity of the Moroccan date palm. It is therefore imperative to assess the genetic diversity and population structure of Moroccan date palms in order to establish strategies for the conservation of this resource and the selection of tolerant and adapted genotypes.

Phenotypic and biochemical markers have been used to assess genetic diversity of date palm, [11–19]. However, those markers are limited, and often affected by environmental conditions or date palm developmental stage. For this reason, molecular markers have been applied to unmask genetic differentiation. In date palm germplasm, different molecular markers such as amplified fragment length polymorphism(AFLP) [20–25], random restriction fragment length polymorphisms (RFLP) [26, 27], random amplified polymorphic DNA (RAPD) [28–35], fingerprints inter simple sequence repeat (ISSR) [36–40], start codon target (Scot) [41, 42], simple sequence repeats (SSR) [43–55], and single nucleotide polymorphism (SNP) [56] were largely used for diversity assessment. Recently, Directed Amplification of Minisatellite-region DNA (DAMD) markers have been employed, for the first time, to characterize the germplasm of the date palm [2].

Indeed, a fitting molecular marker ought to be significantly heritable, and applicable to any part of the genome and should be sufficiently polymorphic to allow the discrimination of narrowly related genotypes [57]. SSRs and DAMDs meet these criteria. They appear, easy to set up and are also remarkably reproducible. In order to acquire a more thorough knowledge of the genetic organization of Moroccan date palm therefore, in this work, we have used both markers. The use of SSR markers is assumed to be one of the best effective strategies in rating date palm genetic diversity. DAMD has been proven as a powerful tool for studding the genetic diversity. In fact, the combination of DAMD and SSR markers would bestow additional and complementary information to measure date palm genetic diversity and population structure.

Description and genetic diversity assessment are important requirements for genetic improvement approaches, and resource management strategies. Hitherto, little is known about genetic diversity and population structure of Moroccan date palm. This research is conducted to assess genetic diversity and genetic structure present in Moroccan date palm population collected from five important Oases in southern Morocco.

The current study is the first report to my knowledge on the genetic diversity assessment and population structure of 276 date palm collected from 5 different zones of Morocco. Additionally, here we also provide a correlation between Moroccan date palm genotypes and some Iraqi cultivars using DAMD and SSR markers.

## Methods

### Plant material

**Fig. 1:**
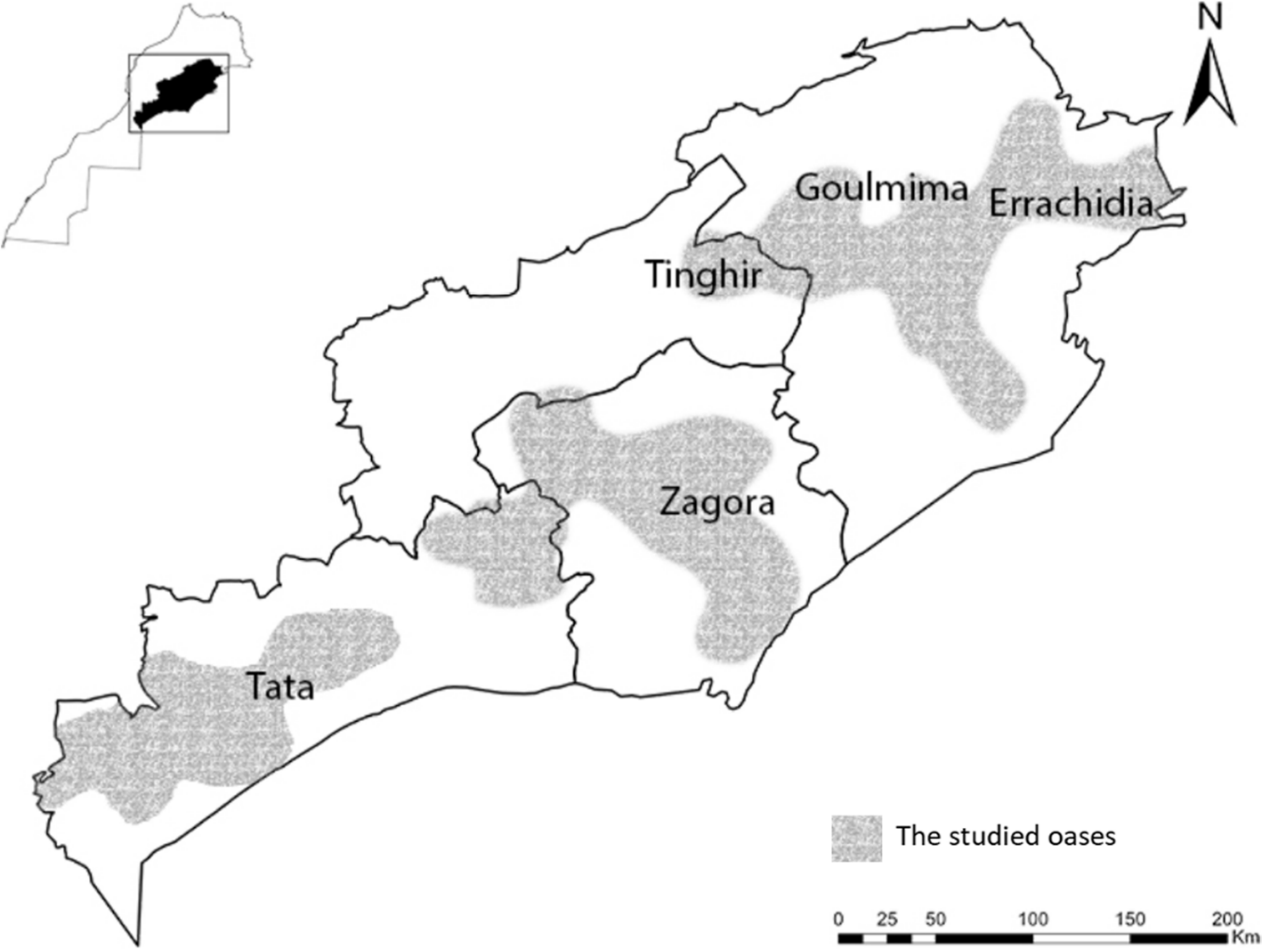
Map of Moroccan sampled regions

A set of 276 date palm leaves were sampled from five important regions of *Phoenix dactylifera* L. population in Morocco: Zagora, Errachidia, Goulmima, Tata, Tinghir. Multiple collection sites were selected from each region (Fig. 1 map). Each location was recorded using a Global Positioning System (GPS) receiver (Additional file 1). The plants were collected to cover the greatest possible genetic diversity in the five traditional regions. The young leaves were selected and picked from healthy adult plants. Then the fresh leaf samples were preserved in a portable refrigerator at – 20 °C and transported to the laboratory for further processing. In addition, we used in this research 10 Moroccan cultivars (five female cultivars contain Majhoul, Boufgouss, Najda, Bouskri, Aziza, Gharass, Sedrat, and three male cultivars that include Nebch-Bouskri NP3, Nebch-Boufeggous NP4, and GS), and one Tunisian accession; Deglet Nour, besides seven Iraqi cultivars (which are female including Maktoum, Khestaoui, Hamrawi, Zahdi, Tebarzal, Bahri, and Habhab).

### DNA extraction

About 60 mg of lyophilized leaves were mechanically ground using a ball mill (Mill: SPEX Sample Prep). Thereafter, DNA was extracted from the ground tissues by using the CTAB (Cetyl Trimethyl Ammonium Bromide) method as described by Saghai-Maroof [58] with minor modifications.

The crushed plant tissue was combined with 1 ml pre-warmed of 2% CTAB buffer (1 M Tris–HCl (pH 8.0), 5 M NaCl, 0.5 M EDTA ethylene diamine tetra acetic acid, 2% CTAB, and 0.2% of β-mercaptoethanol, 0.2% polyvinylpyrrolidone (PVP)), and then the mixture was maintained at 65 °C in a water bath for 60 min; next, the blend was cooled in an ice bucket, and after 700 μl of an equal volume of chloroform isoamyl alcohol (24:1) were added. The tubes which contain the mixture were inverted several times for 15 min to ensure that reagents were mixed well. The mixture was then centrifuged at 13,000 rpm for 10 min.

Next, the supernatant was moved to a new tube and the RNA was eliminated by adding 1 μl of RNA-ase to the mixture, which was incubated at 37 °C for 30 min. Thereafter, 700 μL of ice-cold isopropanol was added to the samples, the mixture was shaken gently and stored overnight to ensure precipitation of nucleic acids.

DNA recovery was completed out by centrifuging at 13,000 rpm for 10 min at 4 °C. and washing twice with ice-cold ethanol (70%). The resulting DNA was dissolved in 200 μL of pure sterile water.

The DNA concentration and quality were assessed by using a NanoDrop spectrophotometer. DNA was deemed pure when the A260/A280 ratio ranged between 1.80 and 2.0 ([53], p. 20). The quality of DNA was also evaluated by electrophoresis, on 1% (w/v) agarose gel. Thereafter all the DNA extracts were stored at – 20 °C.

### SSR analysis

At first, a set of 30 SSR primers were screened on 10 DNA random templates of *Phoenix dactylifera* L. samples. Based on the best amplification results, only 10 SSR primers that gave clear and reproducible bands were eventually chosen for PCR amplification of the entire range of 283 *Phoenix dactylifera* L. genotypes.

The ten polymorphic microsatellites selected were antecedently developed in date palm by [59], the forward and reverse primers of theses markers are presented in Table 1.

**Table 1:** List of 10 SSR primers used in the present study

Primer	Sequences (5′-3′)	Sequences (5′-3′)
	Forward	Reverse
mPdCIR025	F: GCACGAGAAGGCTTATAGT	R: CCCCTCATTAGGATTCTAC
mPdCIR015	F: AGCTGGCTCCTCCCTTCTTA	R: GCTCGGTTGGACTTGTTCT
mPdCIR035	F: ACAAACGGCGATGGGATTAC	R: CCGCAGCTCACCTCTTCTAT
mPdCIR048	F: CGAGACCTACCTTCAACAAA	R: CCACCAACCAAATCAAACAC
mPdCIR050	F: ACAAACGGCGATGGGATTAC	R: CCGCAGCTCACCTCTTCTAT
mPdCIR057	F: AAGCAGCAGCCCTTCCGTAG	R: GTTCTCACTCGCCCAAAAATAC
mPdCIR070	F: CAAGACCCAAGGCTAAC	R: G GAGGTGGCTTTGTAGTAT
mPdCIR085	F: GAGAGAGGGTGGTGTTATT	R: TTCATCCAGAACCACAGTA
mPdCIR090	F: GCAGTCAGTCCCTCATA	R: GCAGTCAGTCCCTCATA
mPdCIR093	F: CCATTTATCATTCCCTCTCTTG	R: CTTGGTAGCTGCGTTTCTTG

The SSR loci’s were performed in a reaction volume of 10 μl consisting of 1 × buffer, 0.5 U of Taq DNA polymerase (BIOLINE, London, UK), 0.5 μM (10 pmol) of each forward and reverse primers and approximately 25 ng of genomic DNA.

The amplification reactions were performed out in the Eppendorf Master cycler with initial denaturation for 1 min at 94 °C. This was followed by 35 cycles of each cycle with 30 s denaturation at 94 °C, 1 min annealing at 52 °C, 2 min extension at 72 °C. and the final extension was carried out at 72 °C for 8 min. The reactions were then held at 4 °C.

### DAMD analysis

Nine minisatellite DAMDs were chosen and used in this study (Table 2) based on their polymorphic information content in published plant diversity analysis researches [2].

**Table 2:** List of 9 DAMD primers used in the present study and their annealing temperatures

Primer names	Primer sequences (5′-3)	Tm (°C)
M13	GAG GGT GGC GGC TCT	60
HBV3	GGT GAA GCA CAG GTG	50
HBV5	GGT GTA GAG AGG GGT	49
HVR	CCT CCT CCC TCC T	40
INS	ACA GGG GTG GGG	40
URP2R	CCC AGC AAC TGA TCG CAC AC	70
URP9F	ATG TGT GCG ATC AGT TGC TG	60
URP25F	GAT GTG TTC TTG GAG CCT GT	59

*Tm(°C)* PCR annealing temperature

The PCR composition was the same as SSR analysis. Polymerase chain reactions (PCR) were performed out according to the program as follows: initial denaturation for 3 min at 94, then 35 cycles of 45 s denaturation at 92 °C, 2 min at annealing temperature (depending on the primer’s Tm), 2 min extension at 72 °C and the Final extension was carried out at 72 °C for 5 min followed by cooling at 4 °C for an unlimited period.

### Gel electrophoresis

In order to detect polymorphism among accessions, the PCR product was transferred to 6% polyacrylamide gel (for higher resolution), using 1 × TBE as running buffer at 120 V. The amplified bands were detected by staining in an ethidium bromide solution for 5 min(10 mg/ml) and visualized under ultraviolet light in Molecular Imager_ Gel DocTM XR System. The gel profiles were photographed and recorded as digital images in a Gel Documentation System for later scoring.

### Statistical analyses

Each visual, clear and distinct band amplified was considered as a single allele and scored manually as present (1) or absent (0) from each SSR and DAMD profiles. Then, a binary data matrix was developed that is used for all analyses.

The effectiveness of each primer used (SSR and DAMD) was assessed by calculating the polymorphic information content (PIC) using the equation $\mathrm{PICvalue}=1-\sum_{\mathrm{n}=1}^{\mathrm{n}}{\mathrm{pi}}^{2}$ where pi is the frequency of the ith allele [60]. Also, (RP) was calculated as a parameter used to detect the ability of each primer to distinguish between individuals, according to Prevost and al [61, 62]. Additionally, the effective marker ratio (EMR) was calculated with the formula EMR = np(np/n), where “np” represents the number of polymorphic loci and "n" represents the total number of loci [63]. Finally, the marker index (MI) was calculated using the formula MI = EMR × PIC, which characterizes the capacity of each primer to detect polymorphic loci among the genotypes.

GenAlex ver. 6.5 software [64] was used to carry out genetic diversity analysis of *Phoenix dactylifera* L. by calculating Nei’s genetic diversity index (H), percentage of polymorphic loci (PPL), number of effective alleles (Ne), expected heterozygoty (He), and Shannon’s information index (I). Molecular variance (AMOVA) was performed, to measure the partitioning of genetic variability between and within populations by the same software. The resulting distance matrices of squared Euclidean distances [65] between all pairwise genotypes, principal coordinate analysis (PCoA) was executed. Based on 999 permutations, the significance of these genetic differentiations was tested. A dendrogram was generated based on Jaccard’s dissimilarity coefficient and UPGMA (Unweighted Pair Group Method of Arithmetic averages) clustering method using XLSTAT 5.14 software.

Finally, to characterize the overall genetic structure and assign individuals to populations, molecular data were analyzed using the STRUCTURE software v.2.3.4 [66] based on Bayesian clustering method. The predefined numbers of populations (K) was set from 1 to 10, and each simulation was performed in 100,000 Monte-Carlo Markov Chain (MCMC) iterations after a burn-in period length of 50,000. The analyzed data resulting from STRUCTURE were then exported to the STRUCTURE HARVESTER online tool [67]. The Evanno approach was applied to determine the most possible structure with the best *K* value.

## Results

### Characterization and efficiency of SSR markers

**Fig. 2:**
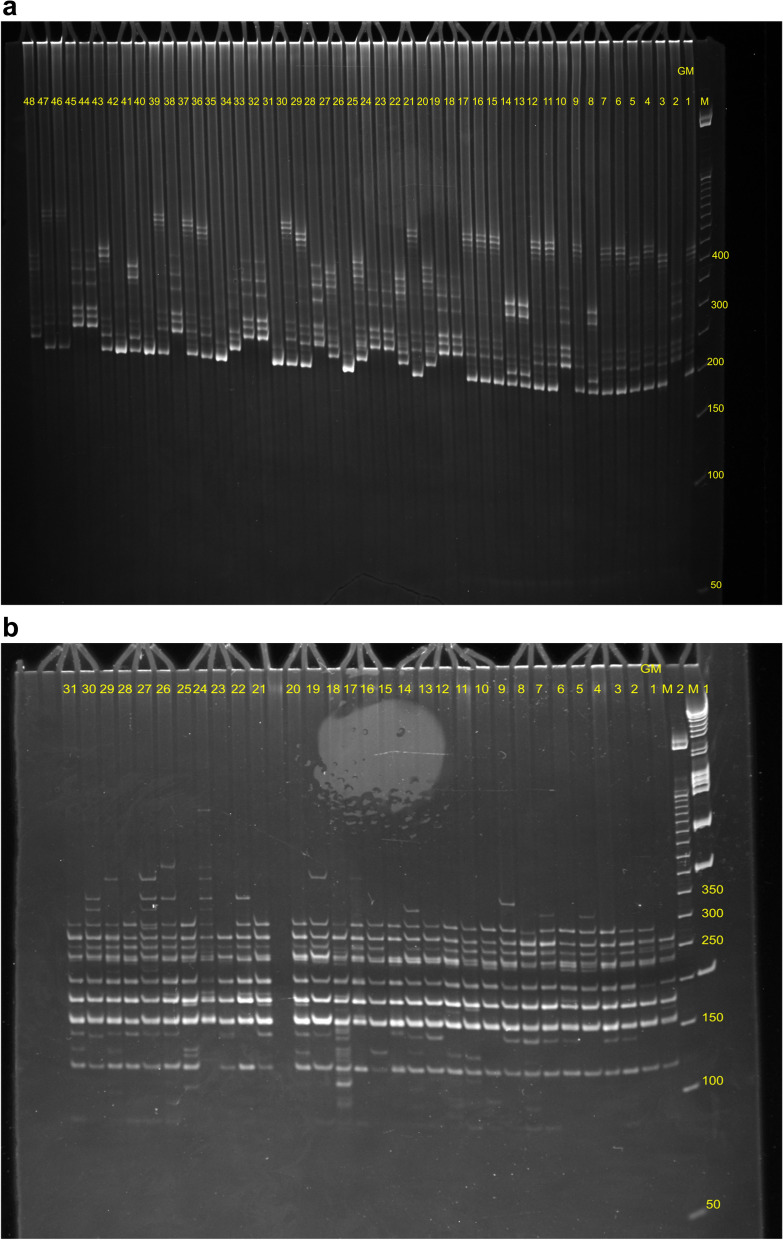
**a** Amplification profile of Cir 25 primer among some Goulmima samples; M, DNA ladder. **b** Amplification profile of URP2R primer among some Goulmima samples; M, DNA ladder

Thirty SSR primers were initially screened for amplification. From these, 10 primers produced sharp, clear, and reproducible banding profile (Fig. 2a) in 283 genotypes of *Phoenix dactylifera* L. The variation of the SSRs amplification frequency based on the availability of motifs in genomic DNA of different genotypes with an average of 24.9 distinct scorable bands were produced by these primers. The primer Cir 50 produced a maximum of 41 scorable bands while the primer Cir 93 generated only 15 fragments. A total of 249 DNA fragments were obtained across all 283 genotypes using the 10 SSR primers (Table 3).

**Table 3:** Efficiency of SSR and DAMD markers in Moroccan *Phoenix dactylifera* L

Primers		TF	MB	PM	PB	PP	PICv	RP(%)	EMR	MI
SSR	Cir 25	26	0	0	26	100	0.992	12.261	26	25.74
	Cir 15	19	4	21.05	15	78.9	0.974	12.869	11.84	11.72
	Cir 35	18	0	0	18	100	0.83	14.424	18	17.82
	Cir 48	18	4	22.22	14	77.7	0.989	20.749	10.88	10.78
	Cir 50	41	0	0	41	100	0.984	18.325	41	40.59
	Cir 57	19	0	0	19	100	0.963	16.304	19	18.81
	Cir 70	29	4	13.79	25	86.2	0.968	17.215	21.55	21.33
	Cir 85	33	0	0	33	100	0.989	17.752	33	32.67
	Cir 90	31	4	12.90	27	87	0.97	12.636	23.51	23.28
	Cir 93	15	0	0	15	100	0.889	13.604	15	14.85
	Total	249	16		233					
	Mean	24.9	1.6	6.99	23.3	92.9	0.954	19.51	21.97	21.76
DAMD	M13	34	0	0	34	100	0.989	33.971	34	33.626
	HBV3	57	0	0	57	100	0.996	24.07	57	56.772
	HBV5	81	0	0	81	100	0.996	36.968	81	80.676
	HVR	45	0	0	45	100	0.987	36.791	45	44.415
	INS	64	0	0	64	100	0.996	36.742	64	63.744
	URP2R	32	0	0	32	100	0.952	16.876	32	30.464
	URP9F	64	0	0	64	100	0.996	20.36	64	63.744
	URP25F	94	0	0	94	100	0.992	29.965	94	93.248
	Total	471			471					
	Mean	58.875			58.875	100	0.98	29.46	58.875	58.33

*TF* Total number of fragments, *MB* Monomorphic bands, *PM* Percentage of monomorphic bands, *PB* Polymorphic bands, *PP* Percent polymorphism, *PICv* Polymorphic information content values, *RP* Resolving power, *EMR* Effective multiplex ratio., *MI* Marker index

The highest number of SSR bands occurred in the Zagora and the Goulmima populations with 232 and 227 respectively, and the lowest number of SSR bands observed in Moroccan varieties and Iraqi varieties. In the latter case, on one private band was observed.

The effectiveness of each primer in tracing polymorphism was evaluated by calculating their PIC and Rp values. PIC value ranged from 0.83 to 0.99, and Rp value ranged from 12.26 to 20.74. The maximum PIC (0.99) and Rp (20.74) values were observed for the Cir 25 and Cir 48 primers, respectively; minimum values for PIC (0.83) and Rp (12.26) were recorded for primers Cir 35 and Cir 25, respectively. In general, the 10 SSR primers utilized in this research recorded an average PIC of 0.954 and an average Rp of 19.51 (Table 4).

**Table 4:** Genetic variation indices and polymorphic features of *Phoenix dactylifera* L. populations revealed by SSR (a) and DAMD (b) markers and combined data of the both markers (c)

a		SSR							
	Populations	N	Na(± SE)	Ne(± SE)	I(± SE)	He(± SE)	uHe(± SE)	%P	Np
	Tinghir	26,000	1,888(0,028)	1,356(0,020)	0,360(0,014)	0,225(0,010)	0,230(0,010)	93,57%	0
	Errachidia	36,000	1,892(0,028)	1,405(0,021)	0,391-(0,014)	0,250(0,011)	0,254(0,011)	94,38%	0
	Tata	52,000	1,984(0,011)	1,408(0,020)	0,402(0,013)	0,255(0,010)	0,258(0,010)	99,20%	0
	Goulmima	55,000	1,952(0,019)	1,369(0,019)	0,381(0,012)	0,238(0,009)	0,240(0,010)	97,59%	0
	Zagora	96,000	1,980(0,011)	1,386(0,019)	0,395(0,012)	0,248(0,009)	0,249(0,009)	98,39%	0
	Moroccan	11,000	1,394(0,058)	1,285(0,020)	0,288(0,015)	0,181(0,011)	0,190(0,011)	68,67%	0
	Iraqi cultivars	7,000	1,414(0,058)	1,364(0,022)	0,338(0,016)	0,220(0,012)	0,237(0,013)	70,28%	1
	Mean		1,786(0,015)	1,368(0,008)	0,365(0,005)	0,231(0,004)	0,237(0,004)	88,87%	
b		DAMD							
	Populations	N	Na(± SE)	Ne(± SE)	I(± SE)	He(± SE)	uHe(± SE)	%P	Np
	Tinghir	26,000	0,934(0,044)	1,243(0,016)	0,216(0,012)	0,143(0,009)	0,146(0,009)	44,56%	16
	Errachidia	36,000	1,037(0,045)	1,243(0,015)	0,229(0,012)	0,149(0,008)	0,151(0,008)	50,31%	18
	Tata	52,000	1,152(0,045)	1,247(0,015)	0,236(0,012)	0,151(0,008)	0,153(0,008)	57,29%	8
	Goulmima	55,000	1,211(0,044)	1,246(0,015)	0,235(0,012)	0,150(0,008)	0,152(0,008)	59,75%	29
	Zagora	96,000	1,349(0,042)	1,254(0,014)	0,252(0,011)	0,160(0,008)	0,160(0,008)	67,15%	39
	Moroccan varieties	11,000	0,690(0,041)	1,148(0,013)	0,139(0,011)	0,090(0,007)	0,094(0,008)	30,39%	0
	Iraqi cultivars	7,000	0,739(0,044)	1,235(0,016)	0,199(0,013)	0,134(0,009)	0,145(0,009)	36,96%	0
	Mean		1,016(0,017)	1,231(0,006)	0,215(0,004)	0,140(0,003)	0,143(0,003)	49,49%	
c	Populations	N	Na(± SE)	Ne(± SE)	I(± SE)	He(± SE)	uHe(± SE)	%P	
	Tinghir	26,000	1,257(0,035)	1,281(0,013)	0,265(0,010)	0,171(0,007)	0,175(0,007)	61,14%	
	Errachidia	36,000	1,326(0,034)	1,298(0,012)	0,284(0,010)	0,183(0,007)	0,186(0,007)	65,22%	
	Tata	52,000	1,433(0,033)	1,302(0,012)	0,292(0,009)	0,187(0,007)	0,188(0,007)	71,47%	
	Goulmima	55,000	1,462(0,032)	1,287(0,012)	0,284(0,009)	0,180(0,006)	0,182(0,007)	72,55%	
	Zagora	96,000	1,563(0,030)	1,299(0,012)	0,301(0,009)	0,189(0,006)	0,190(0,006)	77,72%	
	Moroccan varieties	11,000	0,928(0,036)	1,194(0,011)	0,189(0,009)	0,121(0,006)	0,127(0,007)	43,34%	
	Iraqi cultivars	7,000	0,967(0,037)	1,279(0,013)	0,246(0,010)	0,163(0,007)	0,176(0,008)	48,23%	
	Mean		1,277(0,013)	1,277(0,005)	0,266(0,004)	0,171(0,003)	0,175(0,003)	62,81%	

*N* Number of individuals, *Na* Number of different alleles, *Ne* Number of effective alleles, *I* Shannon’s information index, *He* Expected heterozygosity, *uHe* Unbiased expected heterozygosity, *Np* Number of private bands, *%P* Percentage of polymorphic loci, *(a)* SSR result, *(b)* DAMD result, *(c)* combined result

### Characterization and efficiency of DAMD markers

Wholly eight DAMD markers were reproducible and displayed good banding patterns (Fig. 2b). These markers yielded a varied number of bands. The eight DAMDs in this study produced a total of 471 bands with a mean of 58 (Table 3). The maximum number of DAMD bands were registered for the Zagora population with 330 alleles and Goulmima with 299 bands.

The primer URP25F produced a maximum of 94 bands; URP2R primer produced the minimum of 32 bands. PIC values were calculated for all fragments of DAMD markers which ranged from 0.95 (URP2R) to 0.99 (HBV5 and HBV3) with an average PIC of 0.98. Rp values were evaluated for the eight primers, the lowest Rp value was observed in URP2R (16.87) and the highest one (36.96) in HBV5 with mean of 29.46.

The presence of private alleles is a further indication of population differentiation. Private alleles were detected in Zagora, Goulmima, Errachidia, Tinghir, and Tata (39, 29, 18, 16, and 8 respectively) with DAMD primers, while none were detected in Moroccan and Iraqi varieties (Table 4).

### Genetic diversity

SSR and DAMD markers disclosed various degrees of genetic variability within and between *Phoenix dactylifera* L. populations.

For the SSR markers, the highest values of Genetic parameters viz- observed number of alleles (Na) number of effective alleles (Ne), Shannon’s information index (I), expected heterozygosity (He), and unbiased expected heterozygosity (uHe) were obtained in the Tata population, with values of 1.984, 1.408, 0.402, 0.255, and 0.258, respectively. While the lowest values were recorded in Moroccan varieties, with values 1.394, 1.285, 0.288, 0.181, and 0.190, respectively (Table 4).

For DAMD markers, the data showed that the upper diversity indexes values (Na, Ne, I, He, uHe, and %P) were observed in Zagora population with values 1.349, 1.254, 0.252, 0.160, 0.160, and 67.15% (Table 4). And the lowest values were recorded in Moroccan varieties, with values 0.96, 1.148, 0.139, 0.09, and 0.094, respectively.

Whereas the combination of data from both markers indicated the high genetic diversity indices were recorded in the Zagora population and low values in Moroccan varieties (Table 4).

### AMOVA (analysis of molecular variance) analysis of Phoenix dactylifera L.

**Fig. 3:**
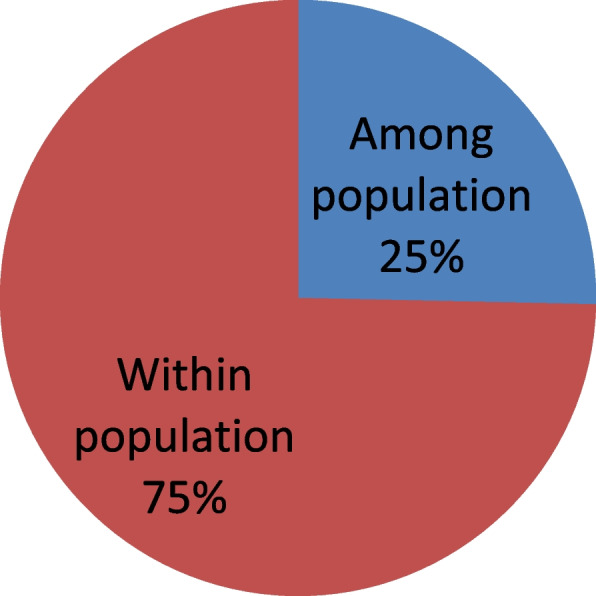
Percentages of molecular variance

AMOVA analysis showed significant variation within population, with 88% for SSR and 65% for DAMD marker (Table 5). While among populations less variance was observed, only 12% and 35% respectively for the both markers. Combined SSR and DAMD data showed 75% of variation within populations and 25% of variations among population (Fig. 3). PhiPT for 283 date palm accessions was 0.253 (*p* < 0.001). Pairwise population PhiPT values for seven clusters ranged from 0.163 (Zagora-Goulmima) to 0.391 (Tinghir-varieties) (Table 6).

**Table 5:** Analysis of molecular variance (AMOVA) of *Phoenix dactylifera* L. from seven regions based on SSR, DAMD and combined data

Marker	Source variation	Df	SS	MS	Est. Var	%
SSR	Among pops	6	1408,835	234,806	5,270	12%
	Within pops	276	10,835,256	39,258	39,258	88%
	Total	282	12,244,092		44,528	100%
DAMD	Among pops	6	5075,386	845,898	21,714	35%
	Within pops	276	11,104,960	40,235	40,235	65%
	Total	282	16,180,346		61,949	100%
Combined SSR and DAMD	Among pops	6	6484,222	1080,704	26,984	25%
	Within pops	276	21,940,216	79,494	79,494	75%
	Total	282	28,424,438		106,478	100%
	Stat	Value	P			
	PhiPT	0,253	< 0.001			

*Df* Degree of freedom, *SS* Sums of squares, *MS* Mean squares, *Est. Var* Estimate of variance, % Percentage of total variation, *PhiPT* Phi-statistics probability level after 1000 permutations, *P* is based on 1000 permutation

**Table 6:** Population PhiPT values based on 999 permutations from AMOVA

	Tinghir	Errachidia	Tata	Goulmima	Zagora	Varieties	Iraq
Tinghir	0.000						
Errachidia	0.275	0.000					
Tata	0.323	0.286	0.000				
Goulmima	0.301	0.283	0.251	0,.000			
Zagora	0.288	0.267	0.193	0.163	0.000		
Varieties	0.391	0.382	0.319	0.294	0.235	0.000	
Iraq	0.335	0.344	0.263	0.320	0.239	0.333	0.000

### Genetic distance

Genetic distance and identity coefficient were evaluated by the method Nei [68].

Based on the combined SSR and DAMD data, the values of the genetic identity were high with the mean genetic similarities among populations ranged from 0.898 to 0.966. In view of the genetic distance, the values vary from 0.035 to 0.108. The Genetic identity and genetic distance between the six regions and varieties found that Zagora and Goulmima populations had the highest genetic identity (0.966), and the lowest genetic distance (0.035; *P* = 0.163). Whereas Errachidia population and Moroccan varieties had the lowest similarity (0.898) and the maximum genetic distance (0.108; *P* = 0.382) (Table 7).

**Table 7:** Pairwise comparison matrix of Nei genetic identity (Above diagonal) and Nei genetic distance (Below diagonal) for *Phoenix dactylifera* L. populations from Tinghir, Errachidia, Tata, Goulmima, Zagora, and Iraq regions based on their geographic origin and *Phoenix dactylifera* L. varieties using SSR and DAMD data

	Tinghir	Errachidia	Tata	Goulmima	Zagora	Varieties	Iraq
Tinghir		0.937	0.923	0.930	0.927	0.903	0.918
Errachidia	0.065		0.935	0.933	0.932	0.898	0.914
Tata	0.080	0.068		0.947	0.958	0.919	0.939
Goulmima	0.072	0.069	0.055		0.966	0.928	0.923
Zagora	0.076	0.071	0.043	0.035		0.938	0.940
Varieties	0.102	0.108	0.085	0.075	0.064		0.918
Iraq	0.085	0.090	0.063	0.080	0.062	0.086	

### Cluster analysis

**Fig. 4:**
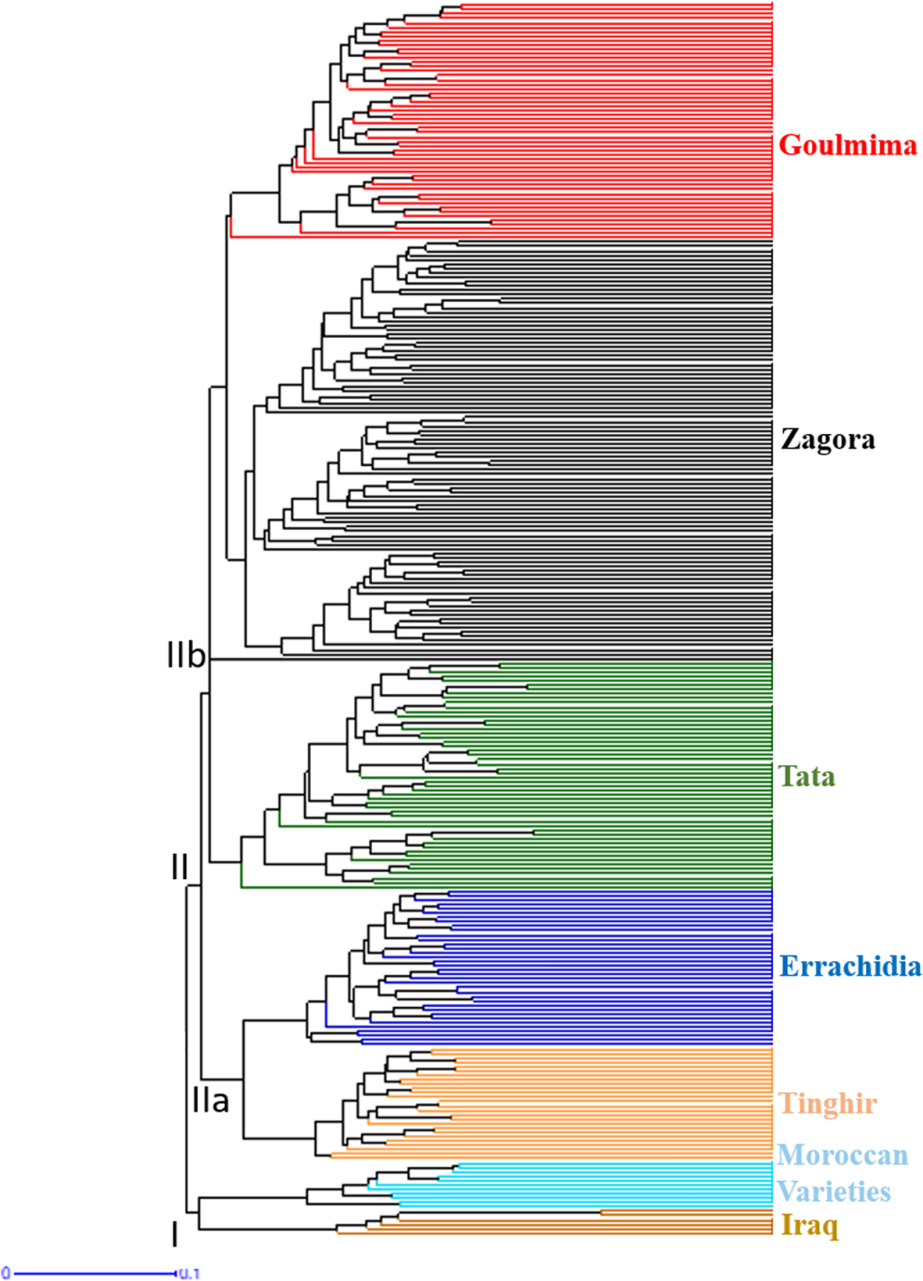
UPGMA cluster study of SSR and DAMD data for all *Phoenix dactylifera* L. individuals sampled

Combined matrix of SSR and DAMD data was employed to build an UPGMA tree. The cluster analysis classified the investigated genotypes into two distinct clusters. The first cluster (I) comprised Moroccan varieties and Iraqi varieties. The second cluster (II) was segregated into two sub clusters. The first one (IIa) comprised of Tinghir and Errachidia populations, and the second sub-cluster (IIb) was split also into two groups, the first group contained Tata population and the second Group encompassed Zagora and Goulmima populations. The UPGMA tree showed in the majority of the individuals were separated according to their populations (Fig. 4).

### Principal coordinate analysis (PCoA)

The PCoA allowed the study of the correlation between different *Phoenix dactylifera* L. genotypes studied. Depending on combined data from SSR and DAMD markers, PCoA illustrated 17.43% of the total variations, with the first three axes (1, 2, and 3) accounted for 7.3%, 5.75%, and 4.38%, respectively.

**Fig. 5:**
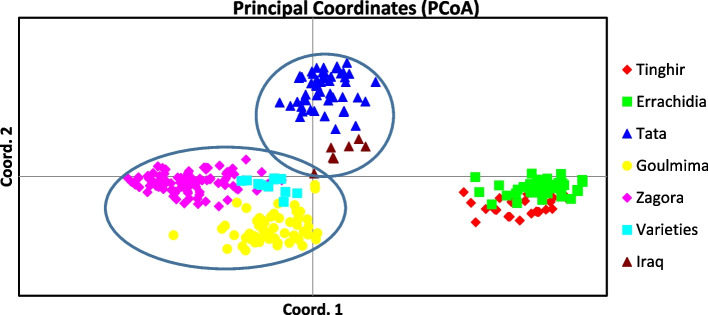
Principal coordinate analysis (PCoA) of the 283 *Phoenix dactylifera* L. genotypes from 5 populations and varieties (Moroccan cultivars) and Iraq cultivars based on combined data from SSR and DAMD markers

The screen plot generated by PCoA highlighted, the grouping of Tata population with Iraqi varieties. The rally of Zagora population with Goulmima population and Moroccan varieties and one genotype from Iraq and the closeness of Errachidia population with Tinghir population (Fig. 5).

### Population genetic structure

**Fig. 6:**
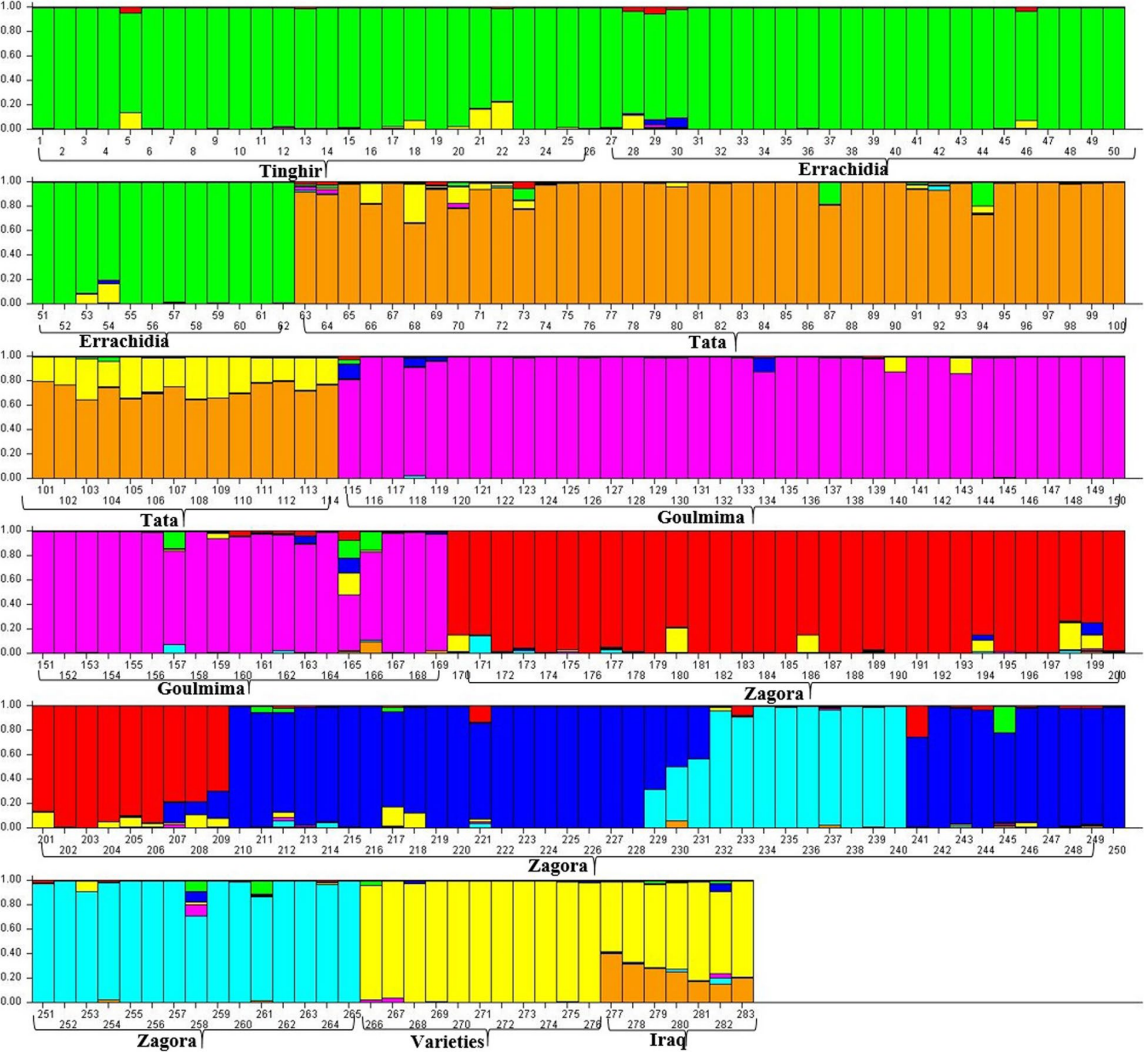
Population genetic structure of 283 *Phoenix dactylifera* L. genotypes at *K* = 7 based on SSR and DAMD data

To characterize the overall genetic structure, we applied a Bayesian clustering approach implemented in STRUCTURE software. The output was extracted with Structure Harvester, following the method of (Evanno et al. 2005) for the estimation of the most likely number of genetic clusters (K), we investigated the range from *K* = 1 to *K* = 10. The maximum value of Δk was detected at *K* = 7. This suggested that the most likely structure of tested samples is their distribution into seven principal clusters (Fig. 6).

Individuals are separated by vertical columns and identified by colors. The same color in different individuals shows that they are part of the same genetic cluster. Different colors in the same individual indicate the likelihood to pertain to different genetic groups.

Our result indicated that the genetic structure of *Phoenix Dactylifera* L. could be grouped into seven populations. The first cluster mainly included of Tinghir and Errachidia population. The second cluster consisted of the Goulmima population. The major group of the Zagora population was split into three clusters. One of the seven clusters was containing the population of Tata and the other genetic cluster comprised Moroccan varieties, while the populations of Iraq varieties were found into both clusters.

## Discussion

For all species evolution and adaptation, genetic variability plays an important role. So, genetic diversity assessment is a key to an effective management and breeding program. Therefore, in this study, we combined SSR and DAMD molecular markers to assess genetic variation among and within *Phoenix dactylifera* L. collection from five Moroccan regions populations.

The results of this investigation revealed that both molecular markers SSR and DAMD generated high polymorphism level with 100% of PP for DAMD primers, and 92.9% for SSR primers. This polymorphism level was higher than that reported by Mirbahar et al. [69] and Srivashtav et al. [70] with 84% and 39.77% polymorphism level using ISSR and RAPD markers respectively. The PIC averages 0.954 and 0.98 obtained using SSR and DAMD markers respectively were informative according to Botstein and al [71]. The SSR primers recorded 19.51 average Rp, while Rp mean value for the DAMD primers was 29.46, showing that DAMD were more discriminant than SSR primers. This data indicates the right choice of primers in the current research for the rating of genetic diversity.

The genotyping data in this study have shown that date palm is rich in allelic variation as confirmed by previous studies [72, 73]. In this research, 249 bands were obtained from 10 SSR primers with a mean of 25 (range from 15 to 41) alleles per locus. This is relatively comparable to an earlier study [74] of date palm from Sudan with female cultivars from Morocco, where the paper reported a total of 343 alleles at the 16 primers with a mean of 21.4 (range from 14 to 44) alleles per locus. A high number of alleles was also detected by al Najm et al. [75] in the assessment of date palm cultivars from Australia and the Middle East by SSR markers which they found 313 bands.

Amplified band number by DAMD were higher than those amplified by SSR markers, indeed 471 bands were amplified with an average of 59. However, the average numbers of each marker in the current study were higher than those reported earlier [53]. The Zagora population had a higher number of private alleles with 39 bands. This result is likely due to Zagora being the largest population and most genetically diverse compared with the other populations.

The genetic parameters detected from the two markers in our findings (SSR: Na = 1,786(0,015) Ne = 1,368(0.008), I = 0.365(0.005) and He = 0.231(0,004) uHe = 0.237(0.004) %*P* = 88.87%; DAMD: Na = 1,016(0.017) Ne = 1,231(0.006), I 0,215(0,004) and He = 0.140(0.003) uHe = 0.143(0,003) %*P* = 49.49%; Combined Na = 1,277(0.013) Ne = 1,277(0.005), I = 0.266(0.004) and He = 0.171(0.003) uHe = 0.175(0.003) %*P* = 62.81%) are a little moderate and higher in SSR. Based on these parameters, the studied populations ranked from the most diverse to the least diverse as follows: Zagora, Tata, Errachidia, Goulmima, Tinghir, Iraqi, and Moroccan varieties. Hence, our findings suggest that the Zagora and Tata populations have the highest genetic variability level. These observations suggest that Zagora and Tata populations could be involved in an effective date palm improvement and conservation strategy.

When assessing genetic relationships of the date palm cultivars using the dominant marker system iPBS Al-Najm et al. [76] noted a Shannon’s Information Index of 0.330, expected heterozygosity of 0.218 and unbiased expected heterozygosity of 0.229). This is in line with the SSR results obtained in the present study (Table 4). Our parameters are higher than the ones obtained from earlier studies in Iranian of date palm cultivars using SCoT molecular markers [42] (Na = 0.671 Ne = 1.179, I = 0.156 and He = 0.104 uHe = 0.114%*P* = 29.67%). While the Al-Najm et al. [75] reported a much higher Shannon’s Information Index (2.067), expected heterozygosity (0.841) and unbiased expected heterozygosity (0.883) in the Australian germplasm when using SSR markers.

Results of AMOVA test have shown that the genetic diversity of Moroccan date palms is highly represented within populations with 75% instead than among populations with 25%. This is in accordance with genetic diversity assessment in date palm genotypes of Sudan [74], and Tunis [77] by SSR markers. Also in Iran using scot [42], SSR and ISSR markers [78], by RAPD and ISSR markers [79], in Morocco by using isozyme markers [80].

The presence of genetic variability within populations is a condition for adaptation and evolutionary transition. Despite date palms are mostly vegetative propagated, most genetic variations observed are essentially caused by mutations, but in the other few cases through sexual reproduction, the meiosis and fertilization lead to immense genetic diversity. Moreover, the famer management had an important impact on this diversity.

However, the Nei analysis revealed low distance values between populations this is in line with AMOVA result which indicate the low diversity among populations with 25%. This finding can be explained by the same origin gene pool of Moroccan date palm. Indeed, Zehdi et al. [81] revealed a deep genetic structuring arising from two geographic gene pools, which means the existence of two cultivation origins of date palm, one in the east and other in the western. Morocco gene pool could belongs to the second region. In contrast, high genetic diversity observed within populations can be result of cloning process, seed reproduction, or natural mutation. This in agreement with Elmeer et al. [82] who reported high genetic diversity observed within the groups and the weak clustering of the cultivars suggested that they are not a result of a full cloning process.

Significant values of similarities were obtained mostly between Zagora and Goulmima populations, as illustrated in the UPGMA dendrogram (Fig. 4), where the two populations are in the same cluster. Sedra et al. [35] reported that most of the cultivars from Iraq were correlated with accessions already grown in Morocco. That agrees with our PCoA pattern which showed that the Tata population and the Iraqi varieties belong to the same group which has already been proven by the high genetic identity of 0.953, also in keeping with our UPGMA which revealed that the Iraqi and Moroccan varieties are in the same cluster that has been verified by similarity value of 0.918. Our results indicated the presence of higher genetic diversity in the Moroccan date palms oasis populations compared to the Iraqi and Moroccan date palms cultivars, which may be explained by intensive selection operations in breeding program [83].

Genetic variation partition in seven differentiated groups obtained with STRUCTURE regrouped Tinghir with Errachidia populations in the same cluster, and Goulmima in another one. While the latter is geographically closer to Errachidia than Tinghir. This can explained by human activities in exchanging disparate materials [84]. Tata population with Iraqi varieties grouped in the same cluster. This is in line with the PCoA result. Zagora population is sub-structured in three groups that confirm the high genetic diversity.

STRUCTURE and UPGMA cluster results showed that there is geo-graphical conservation of accessions. Genetic structure of date palms obtained in this study can be explained by distance geographical isolation, plant biological nature, and environmental conditions [84]. Furthermore, the history of cultivation may contribute to genotypes geographic conservation.

Zagora and Goulmima populations have the higher genetic diversity. Indeed, Zagora harbor 39 private alleles and Goulmima 23. These populations should be prioritized in conservation strategies.

## Conclusion

This work provided a knowledge about genetic resources in the main distribution area of the Moroccan date palm. The SSR and DAMD markers showed adequate polymorphism and offered appropriate details for the genetic diversity assessment of *Phoenix dactylifera* L. Further, AMOVA of this plant showed a high degree of variation within populations.

Genetic diversity reported in our result is essential for evolutionary adaptation in climate change context and for date palm sustainable breeding purposes. Moreover, our results may be a significant guideline for the collection of core germplasm resources. So, we recommend in-situ and ex situ genetic resources management to assure the conservation of this diversity. The approach also must increase the local community awareness of the suitable farming techniques for *Phoenix dactylifera* L. preserving and conservation resources. Consequently, the sustainability of the oases, especially in the era of climate change, will be ensuring.

## Supplementary Information

**Additional file 1: Table S1.** Latitude and longitude of collected accessions.

## Data Availability

All data generated or analyzed during this study are included in this article.

## Abbreviations

AFLP: Amplified fragment length polymorphism
AMOVA: Analysis of the molecular variance
DAMD: Directed amplification of mini-satellite DNA
DNA: Deoxyribonucleic acid
ISSR: Inter simple sequence repeat
PIC: Polymorphic information content
PCoA: Principal coordinate analysis
PCR: Polymerase chain reaction
RAPD: Random amplified polymorphic DNA
RFLP: Restriction fragment length polymorphism
Rp: Resolving power
SCoT: Start codon targeted
SNP: Single nucleotide polymorphism
SSR: Simple sequence repeat
